# Observation of Lévy distribution and replica symmetry breaking in random lasers from a single set of measurements

**DOI:** 10.1038/srep27987

**Published:** 2016-06-13

**Authors:** Anderson S. L. Gomes, Ernesto P. Raposo, André L. Moura, Serge I. Fewo, Pablo I. R. Pincheira, Vladimir Jerez, Lauro J. Q. Maia, Cid B. de Araújo

**Affiliations:** 1 Departamento de Física, Universidade Federal de Pernambuco, 50670-901, Recife-PE, Brazil; 2 Laboratório de Física Teórica e Computacional, Departamento de Física, Universidade Federal de Pernambuco, 50670-901, Recife-PE, Brazil; 3 Grupo de Física da Matéria Condensada, Núcleo de Ciências Exatas - NCEx, Campus Arapiraca, Universidade Federal de Alagoas, 57309-005, Arapiraca-AL, Brazil; 4 Laboratory of Mechanics, Department of Physics, University of Yaoundé I, Cameroon; 5 Grupo de investigación FIELDS, Universidad de Investigación y Desarrollo, Bucaramanga, Colombia; 6 Grupo Física de Materiais, Instituto de Física, Universidade Federal de Goiás, 74001-970, Goiânia-GO, Brazil

## Abstract

Random lasers have been recently exploited as a photonic platform for studies of complex systems. This cross-disciplinary approach opened up new important avenues for the understanding of random-laser behavior, including Lévy-type distributions of strong intensity fluctuations and phase transitions to a photonic spin-glass phase. In this work, we employ the Nd:YBO random laser system to unveil, from a single set of measurements, the physical origin of the complex correspondence between the Lévy fluctuation regime and the replica-symmetry-breaking transition to the spin-glass phase. A novel unexpected finding is also reported: the trend to suppress the spin-glass behavior for high excitation pulse energies. The present description from first principles of this correspondence unfolds new possibilities to characterize other random lasers, such as random fiber lasers, nanolasers and small lasers, which include plasmonic-based, photonic-crystal and bio-derived nanodevices. The statistical nature of the emission provided by random lasers can also impact on their prominent use as sources for speckle-free laser imaging, which nowadays represents one of the most promising applications of random lasers, with expected progress even in cancer research.

Random lasers^1^,^2^,^3^ (RLs) are a special class of lasers with Lévy-type distributions of intensity fluctuations^4^,^5^,^6^,^7^,^8^,^9^,^10^,^11^,^12^,^13^, which has been recently exploited as a photonic platform for studies of complex systems, such as spin glasses^14^,^15^,^16^,^17^,^18^,^19^,^20^,^21^,^22^. RLs were originally proposed^23^ by Letokhov in the late 1960’s, but their first unambiguous observation was only reported in 1994 by Lawandy and collaborators^24^. Over the last two decades, studies in RLs have grown fantastically, including investigations on a myriad of interdisciplinary systems, from cold-atoms RLs^25^ and biomaterials^26^ to speckle-free laser imaging^27^ and cancer diagnostic^28^,^29^.

In a RL system the gain medium, which can be independent of the scatterer or simultaneously gain and scatterer, provides the optical amplification. If the gain medium is independent, the scattering nanoparticles with random spatial distribution are responsible for the necessary optical feedback^1^,^2^,^3^. The external cavity typical of conventional lasers is therefore not required. RL emission has been analyzed in terms of the optical modes and has been regarded as highly multimode^30^, in which the spectral signature can indicate that such modes are averaged out leading to a smooth spectrum, or can indicate the presence of several modes through narrowband spikes appearing after the threshold is reached^31^.

In this work, we demonstrate both theoretically and experimentally the correspondence between the spin-glass behavior of RLs and the Lévy flight statistics of intensity fluctuations. In the photonic context, the term *spin glass* (SG) means that intensity spectra emitted under identical experimental conditions keep a complex pattern of correlations^15^,^16^,^17^,^18^,^19^,^20^,^21^,^22^, as quantified below. Moreover, the *Lévy statistics* implies strong fluctuations in the emission intensity, with non-Gaussian heavy-tailed distribution^4^,^5^,^6^,^7^,^8^,^9^,^10^,^11^,^12^,^13^.

In ref. 19 a replica-symmetry-breaking (RSB) transition to the SG phase was experimentally reported for the first time in a RL employing a functionalized thiophene-based oligomer (T_5_OC_*x*_) in amorphous solid state with planar geometry. RL emission was obtained by pumping with a frequency doubled pulsed Nd:YAG laser (10 Hz, 6 ns, 1.06 *μ*m). The RL spectrum with several spikes could be interpreted as depicting the modes riding on a broad pedestal around 610 nm, as observed when high-resolution spectral measurements were employed. When a lower spectral resolution was employed, a somewhat smooth spectrum was measured. The authors analyzed the shot-to-shot intensity fluctuations in order to obtain the RSB signature and clearly demonstrated the photonic paramagnetic to SG phase transition.

Here, we advance on the understanding and characterization of the photonic behaviors of RLs by reporting on a remarkable match between the Lévy regime of intensity emission and the critical region of the RSB glassy transition. The experimental results were obtained from a single spectral set of data on the Nd:YBO RL system. Our work thus improves on the clarification of the underlying mechanisms of the fluctuation regimes and photonic phases, which are key factors to the progress of the multiple interdisciplinary applications of RLs^1^,^2^,^3^.

## Theoretical Framework

The complex correspondence between the RSB transition to the photonic SG phase and the changes in the statistics of intensity fluctuations in RLs can be explained within the same framework.

We start by reviewing the theoretical ground for the variety of photonic behaviors displayed by RL systems. In a series of remarkable articles^15^,^16^,^17^,^18^,^19^,^20^,^21^,^22^, a phase diagram for lasers in random amplifying media has been recently built based on the Langevin equations for the complex slow-amplitude modes *a*_*k*_(*t*),


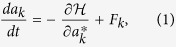


where *F*_*k*_ is a Gaussian (white) uncorrelated noise term and the general complex-valued functional 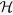 reads (following closely the notation of)^21^


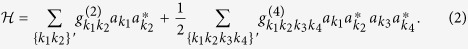


The symbol {…}′ implies the frequency-matching conditions 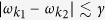 and 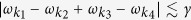 in the quadratic and quartic terms, respectively, with *γ* denoting the finite linewidth of the modes. The physical origin of the quadratic coupling 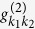 lies in the spatially inhomogeneous refractive index, as well as in a nonuniform distribution of the gain and an effective damping contribution due to the “cavity” leakage. In systems with null or weak leakage in which the off-diagonal contribution is negligible, the real part of the diagonal coupling accounts for the coefficient rates of the amplification (gain) (*γ*_*k*_) and radiation loss (*α*_*k*_) through 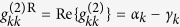. On the other hand, the quartic coupling 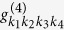 is related to a modulation of the nonlinear *χ*^(3)^ susceptibility with a random spatial profile^15^,^16^,^17^,^18^,^19^,^20^,^21^,^22^.

The spatial disorder generally makes the explicit calculation of the quadratic and quartic couplings in equation (2) rather difficult. In fact, in refs 15, 16, 17, 18, 19, 20, 21, 22 these couplings have been considered as quenched Gaussian variables, with probability distributions independent of the mode combinations {*k*_1_, *k*_2_}′ and {*k*_1_, *k*_2_, *k*_3_, *k*_4_}′, respectively. In a mean-field approach^15^,^16^,^17^,^18^,^19^,^20^,^21^,^22^, all modes are coupled and the frequency-matching restrictions are relaxed. In addition, by considering the total optical intensity, 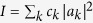, as a constant, with time-independent prefactors *c*_*k*_, the real part 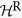 of the functional (2) becomes^21^,^22^ analogue to the Hamiltonian of the *p*-spin model with spherical constraint^32^, which is given by a sum of quadratic (*p* = 2) and quartic (*p* = 4) terms with Gaussian-distributed couplings. It is important to notice that in the photonic-to-magnetic analogy the excitation (pump) energy plays the role of the inverse temperature. An equilibrium statistical physics approach, with the replica trick applied to 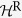 in terms of the slow-amplitude modes *a*_*k*_, then led^21^,^22^ to a phase diagram for the pumping rate as a function of the disorder strength. Photonic paramagnetic, ferromagnetic, phase-locking-wave and RSB spin-glass phases have been characterized^21^,^22^, depending on the trend of the disorder to hamper the synchronous oscillation of the modes. A photonic order parameter, analogue to the Parisi order parameter in SG theory, was suitably defined (see below), so that the value at which it is maximum assumes *q*_max_ = 0 in the prelasing replica-symmetric paramagnetic regime and *q*_max_ ≠ 0 in the random-lasing RSB glassy phase. As a consequence, the RL threshold became identified with the RSB phase transition to the photonic SG phase^15^,^16^,^17^,^18^,^19^,^20^,^21^,^22^.

We now turn to the discussion on the statistical regimes of intensity fluctuations of RL systems in disordered nonlinear media. Noteworthy, the set of Langevin equations, given by equation (1), also provides the underlying theoretical basis for such analysis. Indeed, by writing *I*_*k*_ = *c*_*k*_|*a*_*k*_|^2^, manipulation of equation (1) leads to


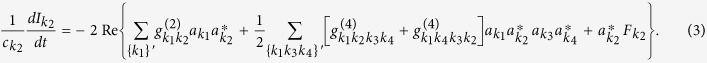


The restricted sum in the quartic coupling generally involves three classes of mode combinations^15^,^33^: 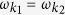 and 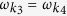, 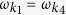 and 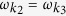, and the remaining possibilities satisfying the frequency-matching condition, which have been usually disregarded^15^,^33^. We consider the diagonal contribution in the quadratic coupling to dominate over the off-diagonal part. By expressing the optical noise as the sum of additive and multiplicative statistically independent stochastic processes^34^, so that 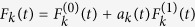, and considering slow-amplitude modes *a*_*k*_(*t*) (if compared to the rapidly evolving phase dynamics), we obtain the Fokker-Planck equation^13^,^34^ for the probability density function (PDF) of emission intensity


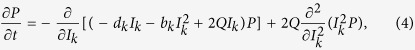


where the parameter *Q* controls the magnitude of the multiplicative fluctuations through 
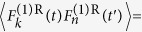
 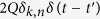, 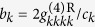, and


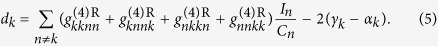


We notice that, by averaging out the rapidly evolving phases to obtain equation (4), the so-called free running approximation has been employed in which the dynamics of the phases and amplitudes are considered as eventually decoupled^15^. In this context, some features of the original model are removed, such as the phase-locking regime^20^,^21^,^22^. Also, in a statistical physics approach we observe that integrating out irrelevant degrees of freedom should be properly done in the partition function, rather than in the Hamiltonian level, though the technical difficulties involved are rather challenging. The steady-state solution of equation (4) is^13^,^34^


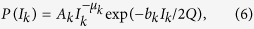


with *I*_*k*_ > 0, *A*_*k*_ as the normalization constant, and the power-law exponent *μ*_*k*_ = 1 + *d*_*k*_/2*Q*.

At this point we remark that, as the statistics of the sum of *N* independent random variables *x* with power-law distribution *P*(*x*) = *Ax*^−*μ*^, 1 < *μ* < 3, is described^35^ by the *α*-stable Lévy distribution with *α* = *μ* − 1, the PDF (6) thus identifies an exponentially-truncated Lévy-like distribution of intensities for 1 < *μ*_*k*_ < 3 and 0 < *α* < 2. In this case, strong fluctuations in *I*_*k*_ emerge and the ultraslow convergence to the *α* = 2 Gaussian behavior due to the presence of the exponential factor in equation (6) is achieved only for a remarkably large *N*^12^,^35^,^36^. In contrast, for *μ*_*k*_ ≤ 1 or *μ*_*k*_ ≥ 3 the central limit theorem assures fast convergence to the *α* = 2 Gaussian statistics of weakly-fluctuating intensities. Therefore, for a given disorder strength, an increasing pumping rate (or excitation pulse energy) raises^13^ the value of *μ*_*k*_ and the statistics of emission intensities shifts progressively from an initial Gaussian (*μ*_*k*_ ≤ 1, *α* = 2), to a Lévy-like (1 < *μ*_*k*_ < 3, *α* = *μ*_*k*_ − 1), and to a subsequent Gaussian (*μ*_*k*_ ≥ 3, *α* = 2) regime.

The correspondence with the RSB glassy transition relies foremost in the recent proposals^6^,^7^,^8^ that assign the Lévy index *α* as an identifier of the RL threshold, which, as discussed, can be also determined by the parameter *q*_max_. Indeed, the above description matches accordingly with recent results on RL systems which report on^7^,^8^,^9^,^10^,^11^,^12^: (i) a prelasing weakly-fluctuating Gaussian regime at low pump energies, corresponding in refs 15, 16, 17, 18, 19, 20, 21, 22 to the photonic paramagnetic (or even the phase-locking-wave) phase with *q*_max_ = 0; followed by (ii) an abrupt change in α at the RL threshold to the strongly-fluctuating Lévy-like regime at intermediate pump energies, signaled in^15^,^16^,^17^,^18^,^19^,^20^,^21^,^22^ by the RSB transition to the glassy regime with *q*_max_ ≠ 0; and (iii) a subsequent crossover at high pump energies to the so-called self-averaged RL regime, with *q*_max_ ≠ 0 and Gaussian statistics of emitted intensities^7^,^8^,^9^,^10^,^11^,^12^. Noticeably, this latter Gaussian regime taking place deep in the glassy RL phase has not been anticipated in refs 15, 16, 17, 18, 19, 20, 21, 22, since the scope of these works did not include the analysis of the statistics of intensity fluctuations.

## Experimental Results and Discussion

The experimental investigation on the above-discussed correspondence is possible through measurements in actual RL systems of the parameter *q*_max_, whose behavior identifies the boundary between the prelasing paramagnetic and RL glassy phases, and the Lévy index *α*, that defines the statistics of the intensity fluctuations as being Gaussian or Lévy-type. As both phenomena arise from the same theoretical framework, we employed the same set of measurements, namely the spectral intensity fluctuations, to obtain both quantities through a proper analysis.

The RL system investigated in this work consisted of crystalline powders of Nd^3+^-doped YBO_3_ (Nd:YBO) – Nd^3+^ concentration: 4.0 mol%^37^,^38^,^39^. The nanoparticle scatterers also act as the gain media. One advantage to work with this solid-state material, in comparison with the use of colloidal systems, is that in the present case the scatterers positions do not change from shot to shot, thus allowing fairly identical experimental conditions to hold over many subsequent excitation pulses^22^. Using a coherent backscattering setup, the measured value of the mean-free-path of photons was much larger than the typical emission wavelength (see Methods).

**Figure 1:**
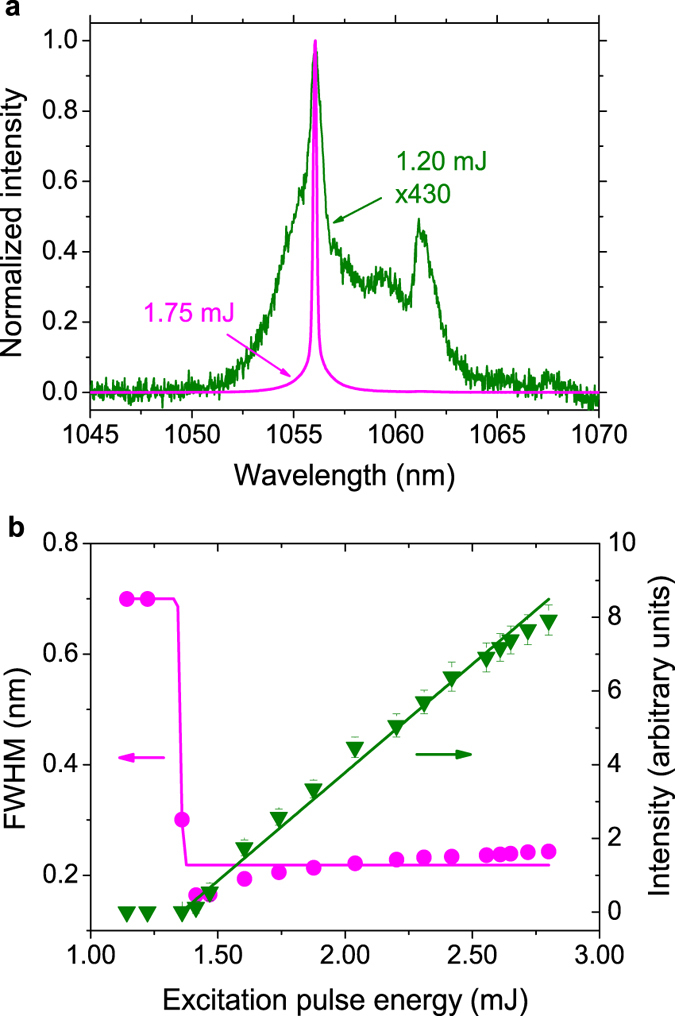
Intensity spectra and the RL threshold. (**a**) Spectral emission of the Nd:YBO system for two excitation pulse energies: below (green, 1.20 mJ) and above (magenta, 1.75 mJ) the RL threshold. (**b**) Emitted intensity (green) and bandwidth narrowing (FWHM, magenta) versus the excitation pulse energy. The intensity measure of the RL threshold implies 1.36 mJ, in close agreement with the FWHM value (1.40 mJ).

Figure 1a displays the spectral emission of the Nd:YBO system for excitation pulse energies below (1.20 mJ) and above (1.75 mJ) the RL threshold, with the latter showing the smooth signature indicating that the modes are averaged out. Even though the emitted RL spectrum is very narrow, as typical of rare-earth-doped RLs^37^,^38^,^39^, it does not indicate single mode operation^31^. In fact, it has been recently shown that even for an ultranarrow smooth spectrum, as those emitted from random Raman lasers^40^, the emission is multimode, as reported in^41^. Actually, the operation in the multimode regime is essential to the preceding theoretical analysis. The emission intensity and bandwidth narrowing are shown in Fig. 1b. With basis on the input-output measurement the estimated RL threshold is 1.36 mJ, which closely agrees with the value 1.40 mJ determined from the full width half maximum (FWHM) of the emitted spectrum. The error bar in the energy measurements is less than 7%.

The characterization of the photonic RSB glassy transition requires the definition^15^,^16^,^17^,^18^,^19^,^20^,^21^,^22^ of an overlap parameter *q*_*γβ*_ analogue to the Parisi overlap parameter in SG theory^14^. Two-point correlations can be calculated either among mode amplitudes *a*_*k*_^20^,^21^, phases^15^,^16^,^17^,^18^ or intensities *I*_*k*_ ∝ |*a*_*k*_|^2 ^19,22, though the latter are the only ones accessible experimentally. In particular, by measuring fluctuations in the spectral intensity averaged over *N*_*s*_ shots (or system replicas; see below), the overlap parameter reads^19^,^22^.


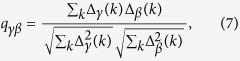


where *γ*, *β* = 1, 2, …, *N*_*s*_, with *N*_*s*_ = 200 at each excitation pulse energy, denote the replica labels, the average intensity at the wavelength indexed by *k* is 
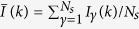
, and the intensity fluctuation is given by 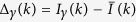. The sum in *k* generally runs over 1100 values, which correspond to the discretization of the spectra in bins of 0.024 nm (see also Methods). Above the threshold, the spectra are narrowed to a FWHM around 0.20 nm, as observed in Fig. 1. In this range one has around 25 non-null values of *I*_γ_ (*k*) for each replica, at each excitation pulse energy. A shot of the pumping laser defines a *replica*, i.e. a copy of the RL system under identical experimental conditions. The PDF *P (q*), equivalent to the Parisi order parameter in the RSB theory of SGs, describes the distribution of replica overlaps *q* = *q*_*γβ*_, signaling a photonic uncorrelated paramagnetic or a SG phase if it peaks exclusively at *q* = 0 (no RSB) or also at values |*q*| ≠ 0 (RSB), respectively.

**Figure 2:**
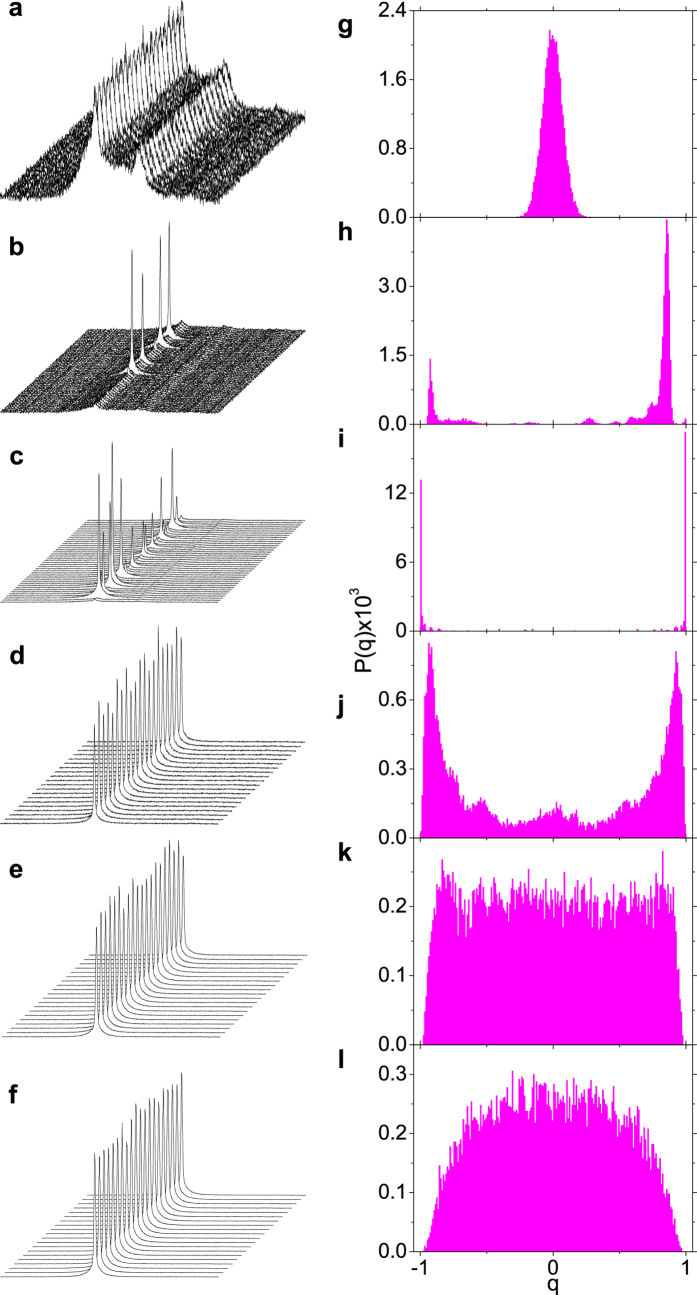
Pulse-to-pulse intensity fluctuations and corresponding overlap distributions signalizing the photonic RSB glassy transition. (**a**–**f**) Intensity spectra showing the fluctuations from shot to shot of the Nd:YBO system for excitation pulse energies (**a**) 1.20 mJ (below the RL threshold), (**b**) 1.36 mJ, (**c**) 1.4 mJ (both around the threshold), (**d**) 1.60 mJ, (**e**) 2.20 mJ and (**f**) 2.80 mJ (above the threshold). (**g**–**l**) PDF distributions of the overlap parameter corresponding to the data in Fig. 2a–f. Fluctuations are stronger (Lévy-type) close to the threshold, in the critical region of the RSB transition from the prelasing paramagnetic to the saturated RL glassy behavior. As the excitation pulse energy increases well above the threshold, fluctuations decline considerably (Gaussian regime) and the SG behavior tends to be suppressed.

In Fig. 2a–f the pulse-to-pulse intensity fluctuations in the Nd:YBO system can be appreciated, as it evolves from the prelasing (Fig. 2a) to the RL regime (Fig. 2b–f). The corresponding plots of the PDFs *P*(*q*), shown in Fig. 2g–l, reveal a rich phase structure, emerging from the photonic paramagnetic (Fig. 2g) to the glassy RL behavior above the threshold. For excitation pulse energies below and just above the threshold (Fig. 2g–i) the photonic behavior is similar to that described in^19^.

For excitation pulse energies well above the RL threshold, the PDF *P*(*q*) starts to broaden (Fig. 2j–l). This is the first report of such behavior, which is related to the deep entry into the Gaussian statistical regime of intensity emission, as discussed below.

**Figure 3:**
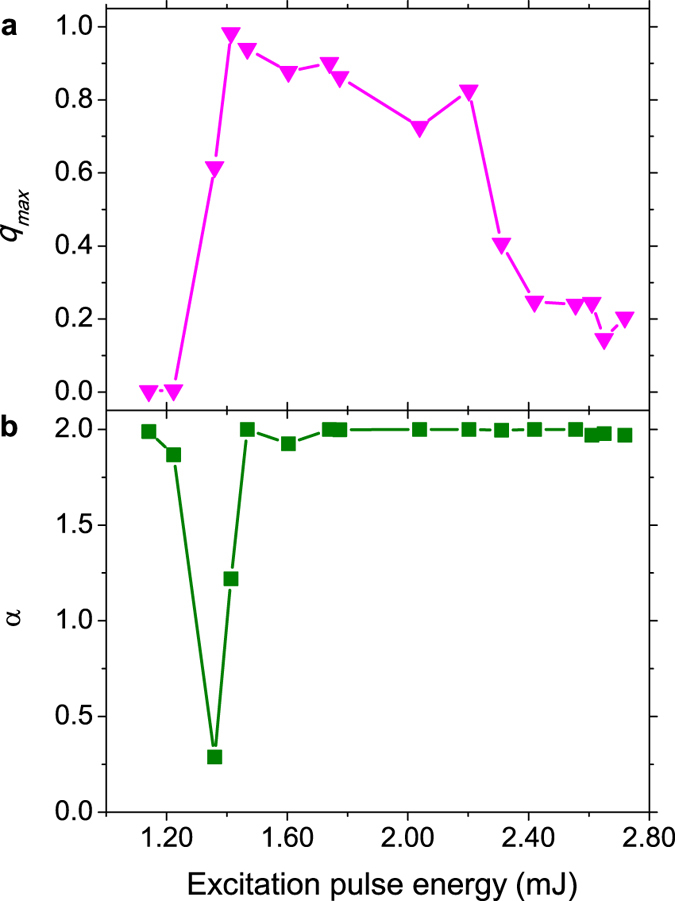
Lévy statistics of intensity emission and the RSB glassy transition. Dependence on the excitation pulse energy of (**a**) the value *q*_max_ at which the Parisi overlap order parameter assumes its maximum and (**b**) the Lévy index *α* calculated from the data in Fig. 2 of the Nd:YBO system. The regime of Lévy statistics (0 < *α* < 2) coincides with the critical region of the RSB transition to the RL glassy behavior. The value *α* = 2 identifies the Gaussian regimes below and above the transition. Notice that well above the threshold the SG behavior tends to be suppressed as *q*_max_ decreases.

The value |*q*| = *q*_max_ at which the distribution *P*(*q*) assumes its maximum is related to the Edward-Anderson order parameter in SG theory^14^,^19^,^22^. Its behavior for the Nd:YBO system is displayed in Fig. 3a, indicating the low-energy prelasing paramagnetic (*q*_max_ ≈ 0), saturated RL SG (*q*_max_ ≈ 1), and high-energy unsaturated RL SG 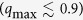 regimes. Differently from the results reported in^19^, however, the behavior of *q*_max_ does not remain nearly constant at the saturation value *q*_max_ ≈ 1, but starts to roll over at high energies, consistently with Fig. 2. Indeed, as seen in Fig. 2e,f, well above the threshold the fluctuations of intensities decline considerably, leading to a decrease in the deviations from the pulse-to-pulse average. Correlations among intensity fluctuations also reduce, causing *q*_max_ to decay.

The above results on the photonic behavior of the Nd:YBO system find an interrelated counterpart in the statistical properties of intensity fluctuations. By analyzing the data in Fig. 2a–f using the quantile-based McCulloch method^8^,^42^, the PDFs of intensities were identified with the family of *α*-stable Lévy distributions, with Lévy index *α* ∈ (0, 2] and boundary value *α* = 2 corresponding to the Gaussian behavior, according to the preceding discussion. By comparing Fig. 3a,b, it is a remarkable fact that, after shifting from the prelasing Gaussian (*α* = 2) statistics, the Lévy (0 < α < 2) regime corresponds to the narrow critical region of the RSB transition from the photonic paramagnetic (*q*_max_ ≈ 1) to the saturated SG RL (*q*_max_ ≈ 1) behavior. Actually, due to the sharp bandwidth narrowing around the threshold, the variation in *α* near the transition is also very acute. Moreover, as the excitation pulse energy increases further a subsequent *α* = 2 Gaussian RL regime sets in, with a trend to suppress the photonic SG phase. Indeed, in this deep Gaussian regime the system presents a considerable weakening of the intensity fluctuations, which also become less correlated as indicated by the decrease in *q*_max_. The existence of a strict causal link between the self-averaged Gaussian regime of intensity fluctuations above the threshold and the observed trend to suppress the glassy phase should be the subject of further studies.

Overall, we remark that the experimental findings on the Nd:YBO system, displayed in Figs 2 and 3, corroborate the preceding theoretical discussion on the correspondence between the photonic RSB glassy transition and the statistics of intensity fluctuations in RLs.

In conclusion, we have analyzed both theoretically and experimentally the physical origin of the complex correspondence between the Lévy flight statistics of intensity emission and the photonic RSB glassy transition in RLs. The experimental demonstration from a single set of spectral measurements on the Nd:YBO RL system indicated a remarkable connection between the behaviors of the Lévy index and the parameter at which the equivalent of the Parisi order parameter is maximum, as a function of the excitation pulse energy. In particular, the Lévy statistical regime of intensities sets in at the RL threshold concurrently with the RSB transition from the photonic paramagnetic to SG behavior. Our results opens up new possibilities to characterize other RLs such as random fiber lasers^43^, nanolasers and small lasers, including plasmonic-based, photonic-crystal and bio-derived nanodevices^44^. Moreover, the statistical nature of the emission provided by RLs can also have impact on their use as sources for speckle-free laser imaging^27^, which is one of the most promising RL applications that can benefit even cancer research^28^,^29^. Finally, these practical aspects are much strengthened by the recent account^45^ showing that the directionality degree of RLs raises to its peak value precisely within the Lévy statistical regime.

## Methods

### Neodymium crystalline powder: preparation and characterization

The random laser system used in this work consisted of crystalline powders of Nd^3+^ doped YBO_3_ (Nd:YBO) obtained by the polymeric precursor method using citric acid (C_5_O_7_H_8_ Sigma-Aldrich) as a complexing agent, d-sorbitol (C_6_O_6_H_1_4, Sigma-Aldrich 98%) as a polymerizing agent, and yttrium nitrate hexahydrate (Y(NO_3_)_3_.6H_2_O, Sigma-Aldrich 99.8%), neodymium hexahydrate (Nd(NO_3_)_3_.6H_2_O, Sigma-Aldrich 99.8%), and boric acid (H_3_BO_3_, Ecibra 99.5%) as precursors for Y, Nd and B, respectively.

The synthesis of the material was achieved by dissolving the yttrium and neodymium nitrates in an aqueous solution of citric acid at room temperature. This solution was added to another solution of d-sorbitol and boric acid previously dissolved in water. The obtained solution was then annealed at 150 °C, whereby the polymerization process occurred, forming a dried resin.The molar ratio of citric acid to elements (metals + boron) was 3:1.The citric acid/d-sorbitol mass ratio was set to 3:2. The dried resin was calcinated at 400 °C during 24 h and heat-treated at 900 °C/1 h.

The X-ray diffraction (XRD) measurements were taken with a Shimadzu XRD-6000 X-ray diffractometer with Bragg-Brentano theta-2 theta geometry, at a continuous scan speed of 1°/min, from 5° to 80° with sampling pitch of 0.01°. K α radiation of 1.54059 Å from a Cu tube operating at 40 kV was used. The XRD pattern revealed that the YBO_3_ phase is an hexagonal structure with P63/mmc (194) space group and centro-symmetric.

The samples were characterized structurally using a JEOL JEM 2010 high-resolution transmission electron microscope (HRTEM) operating at 200 keV. TEM, HRTEM and SAED (selected area of electron diffraction) images of the sample displayed well-crystallized nanoparticles with oval and spherical shaped grains. The sizes distribution obtained by measuring 268 particles depicted particles ranging from 40 to 1000 nm, with the highest occurrence lying around 120 nm.

### Determination of the mean-free-path

For the particle size with the highest occurrence, the scattering mean-free-path, 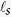, defined as the average distance traversed by a photon between successive scattering events, was measured by a coherent backscattering experiment at 532 nm, using an experimental setup with a cw laser similar to those of refs 46,47.

For the 4.0% Nd:YBO system, the value 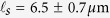 was inferred. In a previous study^48^, the dependence on the particle size of the transport mean-free-path, 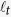, which gives the average distance traversed by photons before changing direction, was measured and calculated (see also ref. 49). For particle sizes around 120 nm, the estimates for 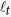, which is close to 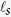, lie in the ranges 5–10 *μ*m and 50–80 *μ*m, at 532 nm and 1056 nm, respectively. Therefore, in either cases the transport mean-free-path is much larger than the typical emission wavelength.

### Optical experiments

The optical experiments were conducted with the powder excited by an Optical Parametric Oscillator (OPO) pumped by the second-harmonic of a Q-switched Nd:YAG laser (7 ns, 10 Hz). The powder was placed on a sample holder and gently pressed into a uniform disc region. The light beam from the OPO was focused on the sample by a 10 cm focal length lens. The illuminated area was 1.8 mm^2^. The excitation wavelength, 806 nm, in resonance with the ^4^I_9/2_ → ^4^F_5/2_ transition, was chosen to optimize the fluorescence signal around 1060 nm due to the ^4^F_3/2_ → ^4^I_11/2_ transition, leading to a RL emission peaking at 1056 nm. The spectra were acquired with a high-resolution spectrometer coupled with a charge-coupled device (CCD), covering, in real time, the range from 1044.563 to 1070.974 nm. In the discretization of the spectra the bin width was 0.024 nm, a value that corresponds to the resolution of the acquisition system.

Although the results presented in the main text refer to the 4.0% Nd^3+^ concentration, we remark that samples with 0.5%, 1.0%, 1.5% and 2.0% were also studied and, except for the 0.5% and 1.0% concentrations in which RL emission was not observed, the other two samples behaved qualitatively in the same way as the 4.0% sample.

## Additional Information

**How to cite this article**: Gomes, A. S. L. *et al*. Observation of Lévy distribution and replica symmetry breaking in random lasers from a single set of measurements. *Sci. Rep.* **6**, 27987; doi: 10.1038/srep27987 (2016).
